# Molecular biomarkers and precision medicine in colorectal cancer: a systematic review of health economic analyses

**DOI:** 10.18632/oncotarget.26909

**Published:** 2019-05-21

**Authors:** Raymond Henderson, Declan French, Richard Sullivan, Tim Maughan, Mike Clarke, Mark Lawler

**Affiliations:** ^1^ Centre for Cancer Research and Cell Biology, Queen’s University Belfast, Belfast, United Kingdom; ^2^ Queen’s Management School, Queen’s University Belfast, Belfast, United Kingdom; ^3^ Institute of Cancer Policy, King's College London and King’s Health Partners Comprehensive Cancer Centre, London, United Kingdom; ^4^ CRUK/MRC Oxford Institute for Radiation Oncology, University of Oxford, Oxford, United Kingdom; ^5^ Centre for Public Health, Queen’s University Belfast, Belfast, United Kingdom

**Keywords:** economic analysis, precision medicine, colorectal cancer, biomarker, KRAS

## Abstract

An increased understanding of the biology of colorectal cancer (CRC) has fuelled identification of biomarkers with potential to drive a stratified precision medicine care approach in this common malignancy.

We conducted a systematic review of health economic assessments of molecular biomarkers (MBMs) and their employment in patient stratification in CRC. Our analysis revealed scenarios where health economic analyses have been applied to evaluate the cost effectiveness of MBM-guided clinical interventions: (i) evaluation of Dihydropyrimidine dehydrogenase gene (DPYD) status to identify patients susceptible to 5-Fluouracil toxicity; (ii) determination of Uridine 5′-diphospho- glucuronosyltransferase family 1 member A1 gene (UGT1A1) polymorphism status to help guide irinotecan treatment; (iii) assessment of RAS/RAF mutational status to stratify patients for chemotherapy or Epidermal Growth Factor Receptor (EGFR) therapy and (iv) multigene expression analysis (Oncotype Dx) to identify and spare non-responders the debilitating effects of particular chemotherapy interventions.

Our findings indicate that Oncotype Dx is cost-effective in high income settings within specific price points, by limiting treatment toxicity in CRC patients. DPYD status testing may also be cost effective in certain settings to avoid specific 5-FU toxicities post treatment. In contrast, current research does not support UGT1A1 polymorphism status as a cost-effective guide to irinotecan dosing, while the health economic evidence to support testing of KRAS/NRAS mutational status and chemo/EGFR therapy choice was inconclusive, despite its widespread adoption in CRC treatment management. However, we also show that there is a paucity of high-quality cost-effectiveness studies to support clinical application of precision medicine approaches in CRC.

## INTRODUCTION

Globally, colorectal cancer (CRC) is the second most common cancer in women (~746,000 new cases annually), and third most common in men (~614,000 new cases annually); and the annual number of deaths approaches 700,000 [1]. CONCORD-3 [2] identified >5.9M CRC patients in 2014. In Europe, 447,000 new cases and 215,000 deaths are reported each year [3]. The economic cost of CRC in the Europe (EU) alone is over €22 billion per year in healthcare costs [Henderson *et al*, manuscript in preparation]. Within the UK, in 2015, almost 42,000 new cases of CRC were documented [4], with over 16,000 CRC deaths [5] and a cost of €2.3 billion (Henderson *et al* Manuscript in Preparation).

An increased understanding of the biology underpinning malignancy has indicated that many cancers, including CRC, are composed of a number of different molecular disease subtypes, which may show differing responses to therapeutic intervention. Identification of appropriate prognostic and predictive molecular biomarkers (MBMs), which can distinguish between these different subtypes, can assist clinical decision-making, such that patients receive the most appropriate treatment based on their molecular profile. This stratified or precision medicine approach has the potential to contribute to enhanced therapeutic efficacy, while minimising treatment-related toxicity.

To identify MBMs of the required clinical utility, e.g. diagnostic (identifying cancer subtype), predictive (determining likelihood of response to therapy), or prognostic (indicating course of disease), analytical platforms are becoming more sophisticated, incorporating technologies such as gene expression profiling and next-generation sequencing. Interpretation of data generated from these platforms is performed using different bioinformatics approaches, adding to overall complexity [6]. The National Institute of Health (NIH) Genetic Testing Registry currently lists 30 MBM tests for CRC [7]. These are employed for a variety of purposes, including: diagnosis, mutation detection/confirmation, pre-symptomatic indications, predictive testing, prognostic determination, drug response evaluation, and treatment management.

For researchers and clinicians to embrace a MBM test, it must demonstrate analytical validity, clinical validity, and, most importantly, clinical utility [8]. These parameters should be established before a cost-effectiveness analysis (CEA) is attempted. Phillips *et al.* [9] examined economic utility analyses of MBM tests in personalised/precision medicine and found that while nearly three quarters of the tests (72%) were associated with better outcomes, these outcomes were in many cases associated with higher costs. However, almost half of the MBM tests fell below a threshold of £35,000 (€40,000 or US$50,000) per quality-adjusted-life-year (QALY), and 20% of the tests showed evidence of cost-savings. A recent paper has identified several MBMs for CRC with prognostic (*BRAF* and DNA mismatch repair status) and predictive (*KRAS* and *NRAS*) utility [10]. Sepulveda *et al.* [10] also note that mismatch repair status indicates a predictive benefit in patients assessed for immunotherapeutic intervention.

Decision makers such as healthcare payers need to know both the financial and the health-related implications of introducing MBM testing. Limited information on the contribution to patient outcomes and societal benefit is often cited as the basis for lack of reimbursement for a particular MBM test [11]. Therefore, the rationale for the systematic review reported here was to compile the body of cost effectiveness evidence generated for MBM testing for CRC in high income health systems, to determine if certain MBM tests can help deliver value-based care.

## MATERIALS AND METHODS

Following Preferred Reporting Items for Systematic Reviews and Meta-analyses (PRISMA) guidelines, this review is registered with PROSPERO (registration number: CRD42016038046) and the findings conform to that registration [12].

### Scoping search

5-Fluorouracil (5-FU) has been the backbone of chemotherapeutic regimens for CRC since the late 1950s. As part of our initial scoping search, we identified thirteen other drugs that have been approved by the Food and Drug Administration (FDA) for treatment of CRC since 1996 (see Supplementary Table 1).

**Table 1:** Screening criteria and study design for systematic review

1	Patients:	Diagnosed with CRC, not limited by age, gender, staging, or type of treatment intervention.
2	Intervention	MBMs including: Single or multi-gene tests (Cobas, Snapshot, Therascreen, High Resolution Melting Assay (HRMA), Sanger sequencing, pyrosequencing, next-generation sequencing, multigene assays, mutational analysis); gene expression profiling (Oncotype DX, Coloprint); protein based tests [immunohistochemistry (IHC)]. All other tests were excluded.
3	Comparator	No MBM test.
4	Outcomes:	The health economic indicator incremental cost-effectiveness ratio (ICER) was investigated, as it relates to cost per QALY and cost per life year gained (LYG).
5	Study design:	Screening for economic analyses based on models (which draw data from trials, resource use and health utility in a disaggregated form) or trials (which prospectively include all the required data). These included CEA, cost-benefit analysis (CBA), cost-minimization analysis (CMA) and cost-utility analysis (CUA). Budget-impact, reviews, letters and editorials were excluded from the systematic review, but were retained for reference.

### Search strategy

Our research question, formulated using the PICOS framework (population, intervention, comparator, outcome, study design) was “What is the cost-effectiveness of using a MBM test for predicting response to therapy in CRC?”. PICOS was employed to develop a search limited to studies that performed economic evaluation of patients diagnosed with CRC, who were subsequently stratified for treatment selection by the result of a MBM test. Initially, a scoping search was performed to identify keywords and MeSH headings. Articles were identified by systematic literature search if they were published between 1 January 2006 and 31 December 2016. We searched MEDLINE, EMBASE, Cochrane Library, SCOPUS, Web of Science, Econlit and SCHARR. Meeting presentations were also searched for the same time period in the American Society of Clinical Oncology (ASCO) and International Society for Pharmacoeconomics and Outcomes Research (ISPOR) websites. Boolean operators were used to set up weekly searches of the above databases throughout the preparation of the review to keep it current, with the addition of Google Scholar alert searches at least 3 times per week until the end of 2018. All bibliographic references retrieved via the searches were exported to reference management software, and duplicates were removed before the study selection step.

### Study selection

Articles were screened for eligibility based on the following criteria (Table 1):

Titles and abstracts of all articles were reviewed for eligibility and only accepted if the above criteria were met. Four reviewers (RH, DF, MC and ML) independently evaluated the full text of potentially eligible articles to determine whether to include these articles in this review. A lack of consensus over eligibility was resolved between the four reviewers. If doubts remained about the suitability of the study (such as academic posters which lack full peer review), we took the conservative approach of including these studies, so as to avoid missing potentially informative studies, while noting that they had not undergone full peer review.

The integrity of each study was assessed according to a checklist developed by the ISPOR Consolidated Health Economic Evaluations Reporting Standards (CHEERS) Task Force Report [13]. This underpinned the development of a quality rating for each study, thus allowing rigorous evaluation of the strength of the data provided. Quality ratings were assigned in five categories: Excellent (✔✔✔✔✔) if a study met 23-out-of 24 CHEERS criteria; good (✔✔✔✔) if 21-22 criteria were met; studies meeting 19–20 criteria were graded as medium (✔✔✔). If only 17–18 criteria were met, the study was graded as low (✔✔), while studies with 16 criteria or less being met were graded as poor (✔).

### Mathematical formulae employed

In cases where more than one therapy were modelled, the reported ICER might not be compared to the base case, e.g. best supportive care (BSC). In these instances, we calculated the ICER based on reported costings and QALYs for the MBM test using the following formula:

$$ICER=\frac{\Delta Costs}{\Delta QALYs}$$

Where LYGs were reported, but not QALYs, and no health utility was reported then:

QALYs=LYGs×0.8

The baseline health utility score of 0.8 was calculated from studies identified in our systematic review, which ranged from 0.71 to 0.87 for progression-free survival in CRC patients, which conforms with a published systematic review of health utility values for CRC [14]. Conversely, where QALYs were reported but not LYGs, and no health utility was reported then:

$$LYGs=\frac{QALYs}{0.8}$$

## RESULTS

### Scoping results

For each therapeutic intervention indicated, we listed the dates of FDA, European Medicines Agency, and National Institute for Health and Care Excellence (NICE) approval. We have identified the annual costs for each of these therapies, adjusted to 2016 £GBP and Euros using the CCEMG (Campbell and Cochrane Economics Methods Group) - EPPI (Evidence for Policy and Practice Information) - Centre Cost Converter [15]. We have also listed putative MBMs for each therapeutic intervention, where applicable, and noted whether each MBM was predictive of therapy, or prognostic of risk (see Supplementary Table 1).

Costs listed in Supplementary Table 1 are drug acquisition costs and do not include costs of outpatient visits, hospitalisation, treatment of side effects, etc. A potential benefit of targeted approaches using biological-based therapy is the avoidance or lessening of adverse effects; e.g. adverse effects resulting from EGFR-targeted therapy are relatively mild (e.g. skin rash) [16] in comparison to those observed with 5-FU (myelosuppression and gastrointestinal toxicity) [17]. It can be difficult to compare targeted therapy directly to chemotherapy as in many instances both are administered in combination, but where targeted therapy is employed, there is potential for a consequential therapeutic benefit in advanced CRC [18]. The MBMs listed in Supplementary Table 1 represent the biomarkers most frequently employed for the indicated therapy.

### Study selection

**Figure 1:**
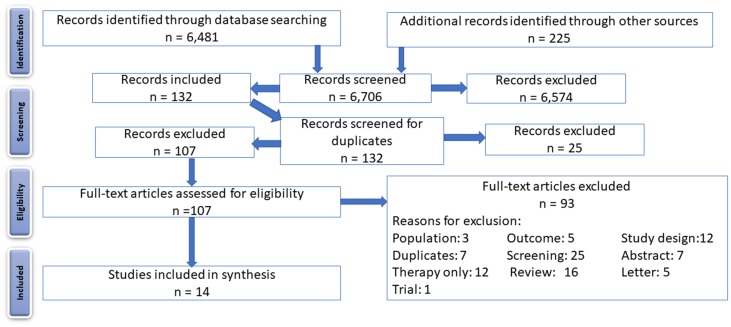
PRISMA flow diagram, showing the flow of identified records through screening, assessment for eligibility, and inclusion.

The Study Selection Workflow is outlined in Figure 1. Our initial database search and other electronic searches (ASCO, ISPOR) followed the search strategy set out in the Methods section and identified 6,706 records. We developed a text-mining algorithm based on health economic filters, as there was a paucity of these represented in the identified records. Consequently, 6,560 records were excluded, and the remaining 146 records were imported into reference management software, where duplicate records (*n* = 25) were removed. A total of 121 articles were then screened for eligibility. After full text examination, 25 articles were excluded as these reported CEAs that related to screening of families for hereditary CRC, which is not relevant to the research question being posed in this systematic review. A further 16 articles were either reviews or systematic reviews which were retained for reference, and 5 articles did not mention the terms LYG, QALY, or ICER. A total of 12 other articles did not include CBAs, CEAs, CMAs, or CUAs and 12 articles focused on CRC therapy alone, not taking into account the use of MBM tests to help guide therapy. On further examination, 7 articles were identified as duplicate studies (earlier abstract reports of the same study or versions of the same study published in other languages), a further 7 were abstracts without sufficient information, 3 articles involved a mixed population of cancer types which either included data already captured or aggregated data from which CRC-specific data could not be extracted, 1 study was an incomplete trial with insufficient data, and 5 were letters with insufficient detail for inclusion. In total, 14 eligible studies remained which involved economic evaluation of a MBM test for guiding therapeutic intervention in CRC.

### Data extraction

We extracted empirical and methodological data and imported the data into Microsoft Excel. Extracted features included: author, year, country of study, CRC stage/metastases/not described, therapy, biomarker utilised, LYG, QALY, and ICER employed (Table 2A–2C). We also extracted Author, perspective (healthcare payer, health insurance or hospital), modelling approach, time horizon (duration of therapy), discounting applied, health utility questionnaire, setting and location, comparisons, scenario analysis, deterministic sensitivity analysis (DSA), and probabilistic sensitivity analysis (PSA) to prepare Table 3A–3C. If there was insufficient data (e.g. abstract reports from conferences), we emailed the original authors for further details.

**Table 2A:** Study characteristics, outcomes, and quality assessment of *DPYD* and *UGT1A1* studies

Author	Year	Country^a^	CRC type	Therapy	Biomarker(Methodology)	LYG	QALY	ICER (£€/QALY)	Quality rating
**Traoré *et al*. [21]**	2012	France	ND	5-FU	*DPYD* (genotyping/ phenotyping)	No	No	£2,795^b^ (€3,175)	✔
**Butzke *et al*. [23]**	2015	Germany	Metastatic	FOLFIRI	*UGT1A1* (PCR)	< 1 day	<1 day	£60,566,870 (€68,810,212)	✔✔✔✔
**Gold *et al*. [24]**	2009	USA	Metastatic	FOLFIRI	*UGT1A1* (PCR)	< 1 day	<1 day	Dominated^d^	✔✔✔
**Obradovic *et al*. [25]**	2008	USA	Metastatic	Irinotecan	*UGT1A1* (PCR)	0.02233	0.01786^c^	£1,318,354 (€1,497,786)	✔✔
**Pichereau *et al*. [26]**	2010	France	Metastatic	FOLFIRI	*UGT1A1* (PCR)	No	No	£966^e^ (€1,097)	✔✔✔

Abbreviations: 5-FU, Fluorouracil; FOLFIRI-FOL, Folinic acid (leucovorin); F - Fluorouracil (5-FU); IRI, Irinotecan (Campto); FOLFOX-FOL, Folinic acid (leucovorin); F, Fluorouracil (5-FU); OX, Oxaliplatin (Eloxatin); ICER, incremental cost-effectiveness ratio; LYG, life year gained; ND, not described; PCR, polymerase chain reaction; QALY, quality-adjusted-life-year.

^a^Country evaluated.

^b^LYG or QALY not stated.

^c^Figures in bold calculated from a 0.8 health utility score.

^d^Dominated; other treatments are less costly and more effective

^e^ICER not based on £/QALY, but as the cost to avoid one febrile neutropenia event per 1000 patients.

A quality rating for each study was determined (see Methods) which allowed us to assign a level of confidence in the strength of evidence for each study. The quality assessment was performed by one reviewer, checked by a second reviewer and any disagreement was resolved by the third and fourth reviewers.

### Data synthesis

Data capture and quality analysis for each study of cost-effectiveness were represented in Tables 2 and 3 and as a narrative summary. Where appropriate, costs were adjusted for year, inflation, and local currency using the CCEMG - EPPI - Centre Cost Converter [15]. The modelling techniques used in the different studies were compared, and their robustness analysed. Finally, the quality of our systematic review itself was checked using two instruments, AMSTAR (Assessing the Methodological Quality of Systematic Reviews) [19] and MECIR (Methodological Expectations of Cochrane Intervention Reviews) [20].

### Study characteristics

Tables 2A–2C outline the characteristics, resulting outcomes, and quality assessment of each study.

#### DPYD

Table 2A lists one study that evaluated *DPYD* gene status in relation to 5-FU toxicity [21]. 5-FU is converted to dihydrofluorouracil by the enzyme dihydropyrimidine dehydrogenase (DPD), expressed by the *DPYD* gene. More than 80% of administered 5-FU is detoxified in the liver by DPD metabolism [22].

#### UGT1A1

Table 2A lists four economic evaluations involving *UGT1A1* testing to guide irinotecan dosing [23–26]. The chemotherapy drug irinotecan is converted principally in the liver by carboxylesterase to SN-38, the active anticancer agent which inhibits topoisomerase I [27].

#### KRAS/BRAF

Table 2B groups the genes *RAS* (*KRAS* and *NRAS*) and *BRAF*, (which are expressed as signalling molecules in the mitogen-activated protein kinase pathway (MAPK)) in relation to their use as prognostic/predictive MBMs in CRC. Tumours harbouring mutated forms of these genes are resistant to anti-epidermal growth factor receptor (EGFR) therapy [28]. *RAS (KRAS and NRAS)* mutations occur in 53% of CRC patients [29] and *BRAF* mutations in approximately 10% [30]. Most of the economic analyses are performed in the context of genotyping the patient for *RAS* mutational status (*KRAS* and *NRAS*) before either treatment with an EGFR monoclonal antibody or chemotherapy treatment.

**Table 2B:** Study characteristics, outcomes, and quality assessment of *BRAF* and *RAS* (*KRAS* and *NRAS*) studies

Author	Year	Country^a^	CRC Type	Therapy	Biomarker (Methodology)	LYG	QALY	ICER (£€/QALY)	Quality rating
**Behl *et al*. [31]**	2012	USA	Metastatic	Cmab Cmab	*KRAS KRAS & BRAF* (PCR clamping and melting curve method)	0.0344 0.0340	**0.0275 0.0272**	£409,877 (€465,633) £396,507(€450,473)	✔✔✔
**Blank *et al*. [32]**	2011	Switzerland	Metastatic	Cmab Cmab	*KRAS KRAS & BRAF* (PCR, then sequencing)	**0.6163 0.6138**	0.4930 0.4910	£49,735 (€56,505) £48,999 (€55,688)	✔✔✔✔
**Carlson JJ [33]**	2010	USA	Metastatic	Cmab	*KRAS* (PCR, then sequencing)	0.1500	0.1100	£204,766 (€232.635)	✔
**Harty, Jarret, and Jofre-Bonet [34]**	2015	UK	Metastatic	Cmab Cmab	*KRAS RAS* (PCR clamping and melting curve method)	0.2900 0.4500	0.2200 0.2400	£73,003 (€82,939) £44,767 (€50.860)	✔
**Health Quality Ontario. [35]**	2010	Canada	Metastatic	Cmab Pmab Cmab + Irinotecan	*KRAS* (Therascreen)	0.3951 0.2903 0.6591	0.3082 0.2264 0.5141	£35,095 (€40,440) £30,607 (€34,773) £27,351 (€31,074)	✔✔✔
**Shiroiwa *et al*. [36]**	2010	Japan	Metastatic	Cmab	*KRAS* (PCR, then sequencing)	0.1800	0.1300	£109,452 (€124,349)	✔✔✔✔
**Vijayaraghavan *et al*. [37]**	2011	USA & Germany	Metastatic	Cmab Pmab Cmab + Irinotecan	*KRAS* (PCR, then sequencing)	0.3804 0.3511 0.4665	**0.3043 0.2809 0.3732**	£58,210 (€66,133) £54,138 (€61,506) £72,714 (€82,611)	✔✔
**Westwood *et al*. [38]**	2014	UK	Metastatic	Cmab	*KRAS* (Therascreen)	**0.2250**	0.1800	£17,616 (€20,013)	✔✔✔✔

Abbreviations: Cmab, cetuximab; EGFR, epidermal growth factor receptor; Pmab, panitumumab.

^a^Country evaluated.

^b^Figures in bold calculated from a 0.8 health utility score.

The majority of health economic evaluations (*N* = 8) involved *RAS* and *BRAF* testing prior to CRC treatment with cetuximab and panitumumab [31–38].

#### Oncotype DX

Table 2C records data associated with the use of a 12-gene assay (Oncotype DX colon cancer) as a predictor of the risk of CRC recurrence [39], thus informing the choice of fluoropyrimidine and FOLFOX (FOL - Folinic acid (leucovorin); F - Fluorouracil (5-FU); OX - Oxaliplatin (Eloxatin)) treatment options.

**Table 2C:** Study characteristics, outcomes, and quality assessment of oncotype DX studies

Author	Year	Country^a^	CRC type	Therapy	Biomarker (methodology)	LYG	QALY	ICER (£/QALY)	Quality rating
**Alberts *et al*. [39]**	2014	USA	Stage II, T3, MMR-P	Fluoro-pyrimidine and FOLFOX	Oncotype DX (12-gene assay RT-PCR)	**0.1425^b^**	0.114	£21,052^c^(€23,917)	✔✔✔✔✔

Abbreviations: MMR-P, mismatch repair proficient; RT-PCR, reverse transcriptase PCR.

^a^Country evaluated.

^b^Figures in bold calculated from a 0.8 health utility score.

^c^Based on a converted US list price of £2,400 for the Oncotype DX colon test.

### Health outcomes for each MBM

#### DPYD

The ICER generated for *DPYD* in Table 2A was £2,795 (€3,175) based on neither LYG nor QALY metrics, but on the prevalence of toxicity episodes from 5-FU, that is, cost per toxicity [21].

#### UGT1A1

The CEAs for *UGT1A1* testing were used to guide irinotecan dosing (Table 2A). The ICERs generated here were all above the NICE threshold of £30,000 (€34,083), with the exception of one study [26], but in this case the ICER was based on numbers of febrile neutropenic events avoided, and not on LYGs or QALYs. Table 2A lists the most significant QALYs generated by these CEAs as being only 6.5 quality-adjusted life days (QALDs) [25]. The two medium and good quality *UGT1A1*/irinotecan CEA studies generated even smaller QALYs, at an average increment that is calculated as less than 2 hours [23] and a decrement of less than two hours [24], which in turn led to the generation of very high ICERs. Additionally, although the Obradovic *et al.* [25] study indicates 6.5 QALDs, we agree with the report from the Centre for Reviews and Dissemination at the University of York [40], that these results should be treated with caution as the protocol was insufficient in detail to allow a quality assessment to be performed

#### KRAS/BRAF

The results in Table 2B indicated that the Health Quality Ontario (HQO) study [35] and the Westwood *et al.* study [38] both yielded ICER values that fell below the NICE threshold. HQO performed three studies, one of which produced an ICER of £27,351 (€31,074), and 187 QALDs using *KRAS* testing prior to cetuximab plus irinotecan treatment, compared to best supportive care (BSC – palliative care). The second HQO CEA for *KRAS* screening prior to panitumumab treatment (compared to BSC) came very close to the NICE threshold with an ICER of £30,607 (€34,773) and over 82 QALDs. The third HQA CEA for *KRAS* screening before cetuximab monotherapy (compared to BSC) generated an ICER above NICE’s threshold at £35,095 (€40,440) with 112 QALDs; however, these 3 studies were rated as medium-quality studies, as the report did not outline a structured summary, describe its analytical methods, or mention funding sources and conflicts of interest. The CEA from the Westwood *et al.* [38] study resulted in an ICER of £17,616 (€20,013) and 66 QALDs and was rated a good quality study in respect to the CHEERS criteria. The other two good quality studies, by Shiroiwa *et al.* [36] and Blank *et al.* [32], produced ICERs of £109,452 (€124,349) (over 45 QALDs for *KRAS* testing prior to cetuximab treatment) and £48,999 (€55,668) (over 179 QALDs for combined *KRAS* and *BRAF* screening prior to cetuximab treatment), respectively. These ICERS were above the NICE threshold of £30,000 (€34,083). The remaining studies were of medium to poor quality, missing important details when applying the CHEERS checklist.

#### Oncotype DX

In Table 2C, the Oncotype DX test [39] for predicting CRC recurrence had a QALY of 0.114, just over 41 QALDs, and the ICER of £21,052 (€23,917) was below NICE’s threshold of £30,000 (€34,083).

### Type of economic evaluation undertaken for each MBM

All studies undertaken were CEAs.

#### DPYD

Incremental costs relating to toxicities following 5-FU administration are indicated in Table 2A [21].

#### UGT1A1

In the CEAs detailing *UGT1A1* genotyping to guide irinotecan dosing, one study [25] reported ICER per LYG, two studies [23, 24] reported ICERs per QALY, while Pichereau *et al.* [26] based their ICER on cost to avoid 1 case of febrile neutropenia per 1000 patients.

#### RAS/BRAF

In Table 2B, 6 of the CEAs involving *RAS* (*KRAS* and *NRAS*) and *BRAF* testing utilised QALYs to calculate their ICERs. The exceptions are Behl *et al.* [31] and Vijayaraghavan *et al*, [37] which employed LYGs.

#### Oncotype DX

In Table 2C, Alberts *et al.* [39] calculated the QALY for the Oncotype DX assay.

### Healthcare perspective, decision model, and time horizon for each MBM evaluated

Tables 3A–3C outline the methods and models used in the included studies.

#### DPYD

Traoré *et al.* [21] did not describe the healthcare perspective for their *DPYD* testing CEA. A decision analytic approach in combination with a Markov model was employed to assess resource use and health outcomes. The time horizon was 2 cycles of chemotherapy.

#### UGT1A1

The healthcare perspective for CEAs for *UGT1A1* genotyping to guide irinotecan dosing was described from the perspective of the healthcare payer in 3 studies [23–25], whereas Pichereau *et al.* [26] focussed on the perspective of the hospital (Table 3A). While Butzke *et al.* [23] employed a decision analytic approach in combination with a Markov model and a lifetime time horizon, the remaining 3 studies solely employed a decision tree to model treatment strategies [24–26], with no specified time horizon (Table 3A).

**Table 3A:** Methodological characteristics of *DPYD* and *UGT1A1* studies

Author	Perspective	Modelling approach	Time horizon	Discount	Health utility	Setting	WTP threshold	Scenario analysis	DSA	PSA
**Traoré *et al*. [21]**	ND	Decision analytic -Markov	2 cycles of chemo-therapy	No	No	ND	ND	No	No	Yes
**Butzke *et al*. [23]**	German statutory insurance	Decision analytic -Markov	Lifetime	3%	EQ-5D	German population	€50,000	No	Yes	Yes
**Gold *et al*. [24]**	Medicare payer	Decision analytic	No	3%	Yes	ND	US$100,000	No	Yes	Yes
**Obradovic *et al*. [25]**	US health-care payer	Decision analytic	No	No	No	ND	US$100,000	No	No	Yes
**Pichereau *et al*. [26]**	French hospital	Decision analytic	No	No	No	Medical care practice in France	ND	No	No	Yes

Abbreviations: DSA, Deterministic Sensitivity Analysis; EQ-5D, EuroQol five dimensions’ questionnaire; PSA, Probabilistic Sensitivity Analysis; WTP, Willingness to Pay.

#### RAS/BRAF

CEA analysis for *RAS* (*KRAS* and *NRAS*) and *BRAF* BM testing to inform anti-EGFR therapy was modelled in 6 of 8 studies from the healthcare payer perspective (Table 2B). All employed a decision analytic approach in combination with a Markov model. Seven (7) of 8 employed a time horizon which varied from 2.5 years to a lifetime horizon (Table 3B), the sole exception being Carlson *et al*. [33], which we ranked as a poor-quality study (missing information included; a structured summary, setting and location, study perspective, time horizon, assumptions made, funding, and conflicts of interest).

**Table 3B:** Methodological characteristics of *BRAF* and *RAS* (*KRAS* and *NRAS*) studies

Author	Perspective	Modelling approach	Time horizon	Discount	Health utility	Setting	WTP threshold in LCU	Scenario analysis	DSA	PSA
**Behl *et al*. [31]**	ND	Decision analytic -Markov	2½ years	3%	No	ND	US$100,000	No	No	Yes
**Blank *et al.* [32]**	Swiss health system	Decision analytic -Markov	Lifetime	3%	HUI3	ND	€40,000	Yes	Yes	Yes
**Carlson JJ. [33]**	ND	Decision analytic -Markov	No	3%	Yes	ND	US$0 to $300,000 CEAC	No	Yes	No
**Harty, Jarret, and Jofre-Bonet [34]**	NHS	Decision analytic -Markov	10 years	No	No	ND	£50,000	No	Yes	No
**Health Quality Ontario. [35]**	Ontario Ministry of Health and Long-Term Care	Decision analytic -Markov	Lifetime	5%	QLQ-C30	Ontario	CAD$50,000	No	No	Yes
**Shiroiwa *et al*. [36]**	Japanese healthcare payer	Decision analytic -Markov	2½ years	3%	HUI3	ND	¥5 to 6 million	Yes	Yes	Yes
**Vijayaraghavan *et al*. [37]**	US & German healthcare payer	Decision analytic -Markov	Lifetime	No	No	ND	ND	No	Yes	No
**Westwood *et al*. [38]**	NHS	Decision analytic -Markov	23 years	3.5%	EQ-5D	England and Wales	ND	No	Yes	No

Abbreviations: HUI3, Health Utility Index Mark 3; QLQ-C30, Quality-of-Life Questionnaire; CEAC, Cost-Effective Acceptability Curve; LCU, Local Currency Units; ND, Not described; NHS, National Health System.

^a^Country evaluated.

#### Oncotype DX

The perspective of the healthcare payer was employed for the Oncotype DX study (Table 3C). A decision analytic approach in combination with a Markov model was employed with a 5-year time horizon (Table 3C).

**Table 3C:** Methodological characteristics of oncotype DX study

Author	Perspective	Modelling approach	Time horizon	Discount	Health utility	Setting	WTP threshold in LCU	Scenario analysis	DSA	PSA
Alberts *et al.* [39]	US third party payer	Decision analytic-Markov	5 years	3%	Yes	Physicians in the MCCRC	US$50,000	Yes	Yes	yes

Abbreviations: LCU, local currency units; MCCRC, Mayo Clinic Cancer Rese arch Consortium.

### Discounting and health utility for each MBM evaluated

Discounting should reflect each country’s borrowing rate, but in health economic analysis the discount rate is usually set by the modellers at between 0% and 7%, with 3% and 5% being the most frequently quoted figures. However, it has previously been reported that almost a third of economic evaluations in healthcare do not use a discount rate [41]. Our findings were similar, with 5 of 14 (36%) studies not using a discount rate.

#### DPYD

A discount rate was not used. No health utility questionnaire was employed.

#### UGT1A1

Two (2) of 4 *UGT1A1* studies [23, 24] employed a discount rate of 3%. These studies also employed a health utility questionnaire; EQ-5D was used by Butzke *et al.* [23] while Gold *et al.* [24] did not specify which health utility questionnaires they employed.

#### RAS/BRAF

Six (6) of 8 *RAS/BRAF* studies employed a discount rate, which ranged from 3-5%. Five (5) of 8 studies employed a health utility questionnaire; in 4 cases [32, 35, 36, 38] the type of health utility questionnaire was indicated (Table 3B)

#### Oncotype DX

The *Oncotype DX* study employed a discount rate of 3% and used a health utility questionnaire, but the type of questionnaire was not specified (Table 3C).

In summary, only CEAs with a good or excellent rating [23, 32, 35, 36, 38] captured the healthcare perspective, decision model, time horizon, discounting, and health utility, the remaining studies were inconsistent, only capturing a number of these parameters. Only 4 studies described a setting and/or location; Pichereau *et al.* [26] and Butzke *et al.* [23] for *UGT1A1 testing*, HQO [35] for *KRAS* testing and Alberts *et al.* [39] for *Oncotype DX* testing. Only Traoré *et al’s DPYD* study [21] used a single study; the remainder were synthesis based. None of the studies covered a reduction in productivity due to adverse effects, illness, or death.

### Willingness to pay thresholds for each MBM evaluated

#### DPYD

A willingness to pay (WTP) threshold was not employed.

#### UGT1A1

Three (3) out of 4 studies reported WTP thresholds, which range from €50,000 in 2013 (£46,950 or €53,340 in 2016) [23] to US$100,000 in 2006 (£81,606 or €92,713 in 2016) [25, 26].

#### RAS/BRAF

Six (6) of 8 studies used a WTP threshold which ranged from a Canadian study [35] which set the threshold at CAD$50,000 (£32,019 or €36,377) to a US study [31] which set the threshold at US$100,000 (£76,440 or €86,844). Carlson [33] constructed a cost-effective acceptability curve (CEAC) with a WTP threshold between US$0 and US$300,000 (£0 or €0 and £232,122 or €263,715) while Westwood *et al.* [38] utilised a CEAC with a WTP threshold up to £100,000 (€111,159). Vijayaraghavan *et al.* [37] did not use a WTP threshold.

#### Oncotype Dx

Alberts *et al.* [39] limited the WTP threshold to US$50,000 (£35,595 or €40,440) (Table 3C).

### Sensitivity analyses for each MBM evaluated

Sensitivity analyses were performed to test the degree of uncertainty in health benefits and costs. DSA tested parameters such as clinical effects, disease progression, QALYs and costs one at a time, while the superior PSA tested these parameters in combination.

#### DPYD

Traoré *et al.* [21] conducted a PSA, but did not detail the findings in their report.

#### UGT1A1

Gold *et al.* [24] and Butzke *et al.* [23] performed both DSA and PSA, whereas Obradovic *et al.* [25] and Pichereau *et al.* [26] only performed PSA. These analyses were employed to address the uncertainty surrounding the cost-effectiveness of irinotecan dosing based on the application of the *UGT1A1* BM test.

#### RAS/BRAF

The PSA in the HQO paper [35] showed that for *KRAS* testing, cetuximab plus irinotecan was the most cost-effective therapy when compared to BSC. The DSA from Vijayaragharan *et al.* [37] indicated that the most sensitive parameter in the model was the percentage of *KRAS* WT patients in the population. The DSA used by Shiroiwa *et al.* [36] did not find parameter changes to have an effect on the results, whilst their PSA found *KRAS* testing to be 62% cost-effective at a WTP threshold of ¥20 million (£196,010 or €222,688). The PSA from Westwood *et al.* [38] did not vary much from the base case, that is, all *KRAS* testing strategies were almost equal.

The scenario analysis performed by Blank *et al.* [31] described a minor impact on the ICER. Blank *et al.* [32] also noted *KRAS* and *BRAF* testing to be the dominant strategy at a WTP threshold of €10,000 to €40,000 (£13,930 to £38,892), whilst at a WTP threshold greater than €40,000 (£38,892), *KRAS* testing was dominant. The tornado plot by Harty *et al.* [34] indicated that the duration in first line progression was most sensitive in the DSA. The DSA performed by Behl *et al.* [31] revealed the model to be sensitive to conversion of chemotherapy costs, cetuximab therapy, and the cost of surgery. Carlson’s [33] DSA detailed via a tornado plot how BSC was most sensitive to difference in QALYs.

Most studies which used a PSA approach [32, 35, 36, 38] came to the same conclusion, namely that it is most cost-effective to use a MBM to test for treatment selection before therapy.

#### Oncotype Dx

The DSA and PSA indicated that QALYs were sensitive to: (1) benefit of fluoropyrimidine monotherapy over surgery alone, (2) benefit of FOLFOX over fluoropyrimidine monotherapy, and (3) time preference discount rate.

### Cost comparison of anti-EGFR therapy versus chemotherapy or BSC

Supplementary Table 2 lists the four studies which included costs of treatment with anti-EGFR therapy compared to chemotherapy (or BSC). For patients with a mutation, health economic analysis indicates that an average of £88,147 (€100,144) per year is saved per patient, due to avoidance of cetuximab therapy based on the *KRAS*/*BRAF* test result. For panitumumab therapy, the average cost saving is £41,159 (€46,761) per patient per year.

### Comparison of ICERs, No MBM versus MBM

Supplementary Table 3A illustrates how the use of MBMs can help increase the cost-effectiveness of treatment, with the exception of *UGT1A1,* which is used to guide the reduction of irinotecan dosing.

As shown in Supplementary Table 3B, the use of the *KRAS* MBM reduced the ICER for cetuximab from £142,515 (€161,912) to £109,452 (€124,349) in the Shiroiwa *et al.* study [36] and for panitumumab from £48,118 (€54,667) to £30,607 (€34,773) in the HQO study [35]. However, although these MBMs improved the cost-effectiveness of these therapies, this did not lead to the intervention achieving an ICER below the NICE threshold in either case, although the HQO study was within the margin of error

### KRAS and BRAF guided chemotherapy costs with corresponding QALYs and ICERs

From reported costs and positive effects (e.g. changes in progression-free survival) in three studies (32, 33, 35), we were able to generate ICERs (Supplementary Table 4) for chemotherapy if the patient population had *KRAS* (or *BRAF*) mutations. Two studies [32, 34] resulted in ICERs below the NICE threshold, at £8,742 (€9,932) and £23,072 (€26,212) respectively. However, the third study by Behl *et al*. [31] while breaching the NICE threshold with an ICER of £49,005 (€55,675), was still under the WTP threshold of US$100,000 (£76,440 or €86,844).

### Genetic heterogeneity within populations and its effect on CEA

It is important to note that the ICERs for the MBMs evaluated in this systematic review are susceptible to the frequency of mutations in the general population. The UGT1A1*28 polymorphism occurs with higher prevalence in the African (42–56%) and Caucasian (26–31%) populations, than in Asian populations (9–16%). Consequently, the use of this biomarker leads to a ten-fold increase in Africans LYGs compared to Asian LYGs, when used to guide irinotecan treatment. However, even with this increase in LYG, this MBM is still not cost-effective [25].

## DISCUSSION

The economic impact of MBM testing to guide therapy in CRC depends upon the cost of the therapeutic intervention and the price of the test, balanced against the clinical impact of the intervention and the degree of toxicity to the patient. So, if the net savings and QALYs are within a specific country’s WTP threshold, the value-based reimbursement of the MBM may help justify a stratified/precision medicine approach to cancer treatment [42].

### DPYD genotyping to guide 5-FU and capecitabine treatment

Deenen *et al.* [43], in a safety and cost analysis of DPYD*2A screening prior to treatment*,* showed that genotyping marginally improves patient outcomes, and is also cost saving (€2,772 screening versus €2,817 non-screening). The CEA by Traoré *et al.* [21] and Deenan *et al.*’s study demonstrates that establishing *DPYD* screening in clinical practice in advance of 5-FU or capecitabine treatment may be cost-effective. On the evidence that we have presented and evaluated in this systematic review, *DPYD* screening is not only cost saving, but also spares patients the associated toxicities, although the overall net monetary benefit may be minimal.

### UGT1A1 genotyping to guide irinotecan treatment

Three of the irinotecan studies [23–25] identified in our analysis suggest that prior testing for *UGT1A1* may be cost saving, but our systematic review is inconclusive as to whether testing improves patient outcomes, with both positive [23] and negative [24] QALYs being reported. Goldstein *et al.* [44] stated that they cannot recommend *UGT1A1* genotyping to guide irinotecan dosing, and that any dose reduction should be based on clinical parameters, rather than *UGT1A1* status.

Lu *et al.* [45] attempted a different approach by escalating the dose in UGT1A1*1 homozygotes and UGT1A1*28 heterozygotes, with positive therapeutic results without the development of adverse effects; a RCT of this approach is ongoing [46]. As the optimum dosing of irinotecan based on *UGT1A1* status has yet to be defined [47], *UGT1A1* genotyping to guide irinotecan dosing will most likely need to be revisited following the availability of results from RCTs such as the one highlighted above in order to determine its efficacy and cost-effectiveness.

### RAS (KRAS and NRAS) and BRAF genotyping to guide treatment

Testing of patients with *RAS (KRAS and NRAS)* and *BRAF* mutations before anti-EGFR or chemotherapy administration has informed clinical decision making in CRC. Of the articles we identified, both the Canadian study by the HQO [35] and the UK study by Westwood *et al.* [38] generate ICERs below the NICE threshold for *KRAS* WT guided therapy compared to BSC and chemotherapy respectively. However, where *RAS* (and *BRAF)* testing were used to select chemotherapy for patients with the mutated form of these genes [32, 34], ICERs were generated below NICE’s WTP threshold, while the study by Behl *et al.* [31] resulted in an ICER below the WTP threshold for the USA. The remaining studies did not report enough information to calculate an ICER for MBM-guided chemotherapy. At present, NICE does not recommend cetuximab as a monotherapy [48] but recommends cetuximab if used in combination with either FOLFOX or FOLFIRI (FOL - Folinic acid (leucovorin); F - Fluorouracil (5-FU); IRI - Irinotecan (Camptosar)) [49]. From the eight studies identified for *RAS* family and *BRAF* mutation testing, the results overall were inconclusive as to whether precision medicine strategies are cost-effective when selecting CRC patients for anti-EGFR therapy. Although NICE’s WTP is set at £30,000, technology appraisals performed for end-of-life treatment guidance, have permitted costs to breach this threshold at an average of £49,000, implicitly suggesting a £50,000 WTP when end-of-life criteria are met. If this figure had been used as our benchmark in this metastatic CRC setting, then half of the studies would be classed as cost-effective [50]. Moreover, when costing and QALY data were reported for selection for chemotherapy treatment, *RAS/BRAF* testing did prove to be cost-effective. The cost savings can be significant. For example, given that more than one million CRC patients in Europe are expected to develop metastatic CRC [2, 51], with 53% harbouring *RAS* mutations, there is the potential to save £3 billion (€3.5 billion) over the lifetime of this patient cohort.

When MBM guided anti-EGFR therapy is compared to anti-EGFR therapy alone, there is a pronounced increase in the ICER values, but the QALYs produced are only marginally different. Thus, *RAS* (and *BRAF*) testing can only be cost-effective when selecting patients who should receive chemotherapy, but not those who receive EGFR therapy, based on the result of their molecular assay.

### Oncotype DX gene assay to guide 5-FU and FOLFOX treatment

The initial economic analysis of the Oncotype DX assay was generated by data from the National Comprehensive Cancer Network and concluded that the assay would improve patient outcomes (QALY = 0.035), and decrease costs by $3,000 for stage II, T3, proficient DNA mismatch repair CRC patients [52]. In the routine practice study by Alberts *et al.* [39], a larger QALY was generated because of the reduction in quality of life associated with adjuvant chemotherapy, and the lower cost savings ($991) due to oxaliplatin coming off patent. The ICER we calculated for the Oncotype DX assay was based on a $3,200 list price [53] and a QALY of 0.114. The applicability of the Oncotype DX Recurrence Score to other populations has been verified in African Americans [54], and although initially inconclusive in the Korean population [55], the larger SUNRISE study has established clinical validity in Asian populations [56]. An economic analysis of the Oncotype DX assay was deemed justifiable and is currently in progress in Israel [57]. Although the Alberts *et al.* CEA [39] was based on only 141 patients, the evidence, in combination with the prior CEA in another study [52], and favourable results from clinical trials [54–56] indicate that the Oncotype DX can spare patients unnecessary chemotherapy, is cost saving and falls below the NICE threshold.

### Previous cost effectiveness analysis of precision medicine approaches in CRC

There have been four previous systematic reviews on the economic analysis of MBM approaches in CRC in the personalised/precision medicine setting. The first was performed by Frank and Mittendorf in 2013 [58]. They identified 7 articles, 3 of referred to the use of the *UGT1A1* MBM, while the remaining 4 investigated the *KRAS* MBM. We captured all 7 of these articles but excluded the study by Mittman *et al.* [59] because it did not utilise a MBM-based approach. Frank and Mittendorf concluded that the cost-effectiveness of *UGT1A1* testing prior to irinotecan administration remains unresolved, whilst using *KRAS* genotyping to stratify patients before anti-EGFR treatment was cost-effective. The second systematic review, by Westwood *et al.* in 2014 [38], was a health technology assessment focusing on the cost-effectiveness of *KRAS* testing of CRC tumours. Its literature search found 5 articles, which we also identified, and the authors concluded that the ICER for *KRAS* mutation testing to guide anti-EGFR therapy was large. However, although they performed a CEA and found *KRAS* testing to be cost-effective, their results should be interpreted with caution as a number of assumptions were made in relation to resection rates, MBM test use, etc. The third paper by Guglielmo *et al.* [60] identified 5 KRAS studies, which we also uncovered in our literature search, but the study by Barone *et al.* [61] which they included did not meet our PICOS criteria as a CEA. Additionally, their review did not include the Canadian [35] or Japanese [36] studies we identified, because their search was limited to 2011 to 2016. Their findings for *KRAS* testing to guide anti-EGFR therapy were inconclusive. The fourth review by Seo and Cairns [62] identified 46 studies but only 12 were relevant to our research question. We captured 11 of these studies, missing a poster by Niedersuess-Beke D. *et al.* [63]. Our findings correspond with Seo and Cairns, in that *KRAS* testing is always more cost-effective, even if this is not always the case for anti-EGFR therapies. However, we draw different conclusions from the data for the irinotecan studies, finding *UGT1A1* testing not to be cost-effective.

We disagree with the Frank and Mittendorf systematic review on the lack of evidence to make a decision on the cost-effectiveness of *UGT1A1*, because our analysis indicates that there is enough evidence to support the assertion that the use of *UGT1A1* genotyping to reduce irinotecan dosing is not cost-effective. Despite being able to select patients to receive chemotherapy, our findings suggest that there is insufficient evidence to indicate *KRAS* (and *BRAF*) testing is cost-effective, in the context of EGFR therapy.

It is evident that not all CRC patients currently benefit from precision medicine MBM-informed therapy, as is the case for the 53% of RAS mutant mCRC patients not eligible for anti-EGFR treatment [29]. The emerging field of molecular pathological epidemiology permits associations to be made between particular exposures (e.g. microorganisms, diet, lifestyle) and molecular pathological responses, identified through research on, for example, the interplay between the microbiome, tumour cells, and the immune system [64]. Patients harbouring *PIK3CA* mutated tumours benefit from exposure to aspirin, whereas *PIK3CA* wild-type patients do not [64]. Microsatellite instability high (MSI-H) tumours provoke a vigorous immunotherapeutic response; however, the presence of *Fusobacterium nucleatum* may counteract MSI-H positivity with associated immunosuppressive effects [64, 65]. The challenge in CEA is how to leverage multiple biomarkers such as *RAS*, *BRAF*, *PIK3CA*, MSI-H, and *F.nucleatum* positivity in a cost effective manner to precisely guide anti-EGFR therapy, aspirin therapy, and immunotherapy in mCRC. This challenge is becoming increasingly relevant as treatment algorithms incorporating multiple biomarkers become more common place and techniques such as whole genome sequencing enters clinical practice.

## CONCLUSIONS

There is a paucity of high-quality CEAs that evaluate MBM in CRC. Unless CEA is incorporated prospectively into clinical trial design, economically unsubstantiated results can obscure the best available evidence, undermining both methodological approaches and resources. In summary, we found that the cost-effectiveness of MBM approaches to guide CRC therapy is highly variable. The evidence from our review suggests that *DPYD* screening could be cost-effective in high-income settings, if it is implemented before 5-FU therapy. Likewise, Oncotype DX assay is likely to be cost-effective in identifying patients who will not benefit from chemotherapy. We were unable to find evidence to support *UGT1A1* testing to guide irinotecan dosing. Perhaps more controversially, despite its adoption in many countries globally, we found that the cost-effectiveness data currently available to support anti-EGFR treatment based on *RAS/BRAF* mutational status is inconclusive.

The evidence presented here reflects a need for a more rigorous methodological CEA-driven approach to be prospectively employed. There also needs to be greater transparency on prices used in CEA, so as to ensure the delivery of value-based care in a disease that kills nearly 170,000 Europeans every year.

## SUPPLEMENTARY MATERIALS



## Abbreviations

CRC: colorectal cancer
MBM: molecular biomarker
DPYD: Dihydropyrimidine dehydrogenase
UGT1A1: Uridine 5′-diphospho- glucuronosyltransferase family 1 member A1
EGFR: epidermal growth factor receptor
5-FU: Fluorouracil
QALY: quality-adjusted lifer year
QALD: quality-adjusted life day
PICOS: population intervention comparator outcome study design
ISPOR: International Society for Pharmacoeconomics and Outcomes Research
CHEERS: consolidated health economic evaluations reporting standards
ICER: incremental cost-effectiveness ratio
BSC: best supportive care
LYG: life year gained
NICE: National Institute for Health and Care Excellence
CEA: cost-effectiveness analysis
DSA: deterministic sensitivity analysis
PSA: probabilistic sensitivity analysis
FOLFOX: FOL-Folinic acid (leucovorin): F-Fluorouracil (5-FU): OX-Oxaliplatin (Eloxatin)
HQO: Health Quality Ontario
WTP: willingness-to-pay
CEAC: cost-effective acceptability curve
MSI-H: microsatellite instability-high
