# Supplementary material for: Molecular biomarkers and precision medicine in colorectal cancer: a systematic review of health economic analyses
Source: Oncotarget. 2019 May 21;10(36):3408–23. doi: 10.18632/oncotarget.26909 (PMC6534362; doi:10.18632/oncotarget.26909)
Supplement: Supplementary file 1 [file oncotarget-10-3408-s001.pdf]

Molecular biomarkers and precision medicine in colorectal cancer: a systematic review of health economic analyses

SUPPLEMENTARY MATERIALS

Supplementary Table 1: CRC treatments and their corresponding BMs

FDA approval [1]	Therapy	Therapy type	Annual cost	NICE approval [2]	EMA approval [3]	Putative biomarker	Biomarker predictive of therapy	Biomarker prognostic of risk	Reference
1962	Fluorouracil	Chemo-therapy	£330 (€375) [4]	Predates NICE	Predates EMA	MSI-H<br>DPYD	 Yes	Yes	[5]<br>[6]
1996	Irinotecan	Chemo-therapy	£7,852 (€8,921) [7]	2002	ND	UGT1A1	Yes		[8]
2002	Oxaliplatin	Chemo-therapy	£18,528 (€21,111) [7]	2002	ND	Oncotype DX		Yes	[9]
2005	Capecitabine	Chemo-therapy	£6,789 (€7,713) [10]	2003	2001	DPYD	Yes		[10]
2004	Bevacizumab	Targeted Therapy	£36,000 (€40,900) [11]	2007	2005	VEGF<br>VEGFR-1	Yes<br>Yes		[12]
2004	Cetuximab	Targeted Therapy	£37,000 (€42,036) [11]	2007	2004	RAS (KRAS and NRAS)<br>BRAF	Yes<br>Yes	 Yes	[13]
2006	Panitumumab	Targeted Therapy	£49,000 (€55,669) [11]	2011 (No)	2009	RAS (KRAS and NRAS)<br>BRAF	Yes<br>Yes	 Yes	[13]
2011	Ipilimumab	Immuno-therapy	£75,000 (€85,208) [14]	In appraisal	Not appraised	MSI-H	Yes	Yes	[15]
2012	Aflibercept	Targeted Therapy	£30,000 (€34,084) [16]	2014 (No)	2013	IL8	Yes		[17]
2014	Nivolumab	Immuno-therapy	£91,000 (€103,385) [14]	In appraisal	Not approved	MSI-H	Yes	Yes	[15]
2015	Ramucirumab	Targeted Therapy	£100,000 (€113,610) [18]	2016	2014	VEGFR-2	Yes		[19]
2012	Regorafenib	Targeted Therapy	£58,000 (€65,894) [20]	2015 (No)	2013	VEGFR-2	Yes		[21]
2015	Pembrolizumab	Immuno-therapy	£86,000 (€97,705) [22]	In appraisal	Not appraised	MSI-H	Yes	Yes	[15]
2015	TAS-102	Chemo-therapy	£26,416 (€30,011) [23]	2016	2016	None presently identified	N/A	N/A	

BRAF - gene for B-raf; DPYD - dihydropyrimidine dehydrogenase; EMA – European Medicines Agency; FDA – Food and Drug Administration; IL8 – interleukin 8; KRAS - kristen retrovirus-associated DNA sequences; MSI-H - microsatellite instability high; NICE – National Institute for Health and Care Excellence; ND – not described; RAS - retrovirus-associated DNA sequences; TAS-102 - Trifluridine/tipiracil; UGT1A1 - UDP glucuronosyltransferase 1 family, polypeptide A1; VEGF - vascular endothelial growth factor; VEGFR-1 - vascular endothelial growth factor receptor 1; VEGFR-2 - vascular endothelial growth factor receptor 2.
Bevacizumab is a monoclonal antibody (MoAb) that targets the protein VEGF blocking angiogenesis.
Cetuximab is a MoAb that targets the epidermal growth factor receptor (EGFR) blocking cell growth.
Panitumumab is a MoAb that targets the EGFR blocking cell growth.
Ipilimumab is a MoAb that targets cytotoxic T-lymphocyte-associated protein 4 (CTLA-4) preventing it from inhibiting the immune response of cytotoxic T cells against cancer cells.
Aflibercept is a recombinant fusion protein that targets the protein VEGF blocking angiogenesis.
Nivolumab is a MoAb that blocks programmed cell death 1 ligand 1 (PD-L1) from binding to programmed cell death protein 1 (PD-1) on the T cell allowing an immune response against the tumour.
Ramucirumab is a MoAb that targets the protein VEGFR-2 blocking downstream angiogenesis.
Regorafenib is a protein tyrosine kinase inhibitor (TKI) with anti-angiogenic activity targeting VEGFR-2.

Pembrolizumab is a MoAb that blocks PD-L1 from binding to PD-1 on the T cell allowing an immune response against the tumour

Supplementary Table 2: Annual cost savings per patient of not using anti-EGFR therapy on KRAS mutated mCRC

Study	Country	Cetuximab drug cost	Panitumumab drug cost	Chemotherapy drug cost	Cost savings of not using cetuximab	Cost savings of not using panitumumab
**Behl *et al*. [24] (2012)**	USA	£267,876 (€304,334)	-	£17,927 (€20,367)	£249,949 (€283,967)	-
**Health Quality Ontario. [25] (2010)**	Canada	£45,047 (€51,178)a	£35,465 (€40,292)[a]	-	£45,047 (€51,178)	£35,465 (€40,292)
**Shiroiwa *et al*. [26] (2010)**	Japan	£43,935 (€49,915)	-	£843 (€958)	£43,092 (€48,957)	-
**Vijayaraghavan *et al*. [27] (2011)**	USA	£87,860 (€99,818)	£70,779 (€80,413)	£8,107 (€9,210)	£79,753 (€90,608)	£62,672 (€71,203)
**Vijayaraghavan *et al*. [27] (2011)**	Germany	£58,421 (€66,372)	£60,866 (€69,150)	£35,527[a] (€40,362)	£22,894 (€26,010)	£25,339 (€28,788)

[a]Data for cost of test not present.

[b]Costs for chemotherapy in Germany are much higher than in the USA because FOLFIRI is used rather than irinotecan monothera

Supplementary Table 3A: Comparison of ICERs without and with Biomarkers for Chemotherapy

		ICER	
**Therapy**	**Test**	**No biomarker**	**Biomarker**
Fluorouracil	DPYD	£11,593 (€13,171) [28]	£2,795 (€3,175) [29]
FOLFIRI	UGT1A1	£20,626 (€23,433) [30]	Not Applicablea
5-FU and FOLFOX	Oncotype Dx	£43,210 (€49,091) [28]	£21,052 (€23,917) [31]

[a]All ICERs for *UGT1A1* genotyping for guidance of irinotecan dosing were in excess of £1 million (€1 million) and therefore disregarded (see Table 2A).

Supplementary Table 3B: Comparison of ICERs without and with biomarkers for targeted therapy

	ICER	
**Therapy**	**No biomarker**	**Biomarker**
Cetuximab	£142,515 (€161,912) [26]	£109,452 (€124,349) [26]
Panitumumab	£48,118 (€54,667) [25]	£30,607 (€34,773) [25]

Supplementary Table 4: *KRAS* and *BRAF* guided chemotherapy costs with corresponding QALYs and ICERs

Study	Country	Chemotherapy cost	QALY	ICER (cost/qaly)
**Behl *et al*. [24] (2012)**	USA	£26,212 (€29,780) [a]	0.5349	£49,005 (€55,675)
**Blank *et al*. [32] (2012)**	Switzerland	£3,873 (€4,400)	0.4430	£8,742 (€9,932)
**Harty, Jarret, and Jofre-Bonet [33] (2015)**	UK	£31,840 (€36,174)	1.3800	£23,072 (€26,212)

[a]Includes costs of resection.

REFERENCES

1. National Institute for Health. A to Z List of Cancer Drugs. Available 2019 Mar 13, from: https://www.cancer.gov/about-cancer/treatment/drugs/.
2. National Institute for Health and Clinical Excellence - NICE. Technology appraisal processes. Available 2019 Mar 13, from: https://www.nice.org.uk/about/what-we-do/our-programmes/nice-guidance/nice-technology-appraisal-guidance/process.
3. European Medicines Agency. What we publish on medicines and when. Available 2019 Mar 13, from: https://www.ema.europa.eu/en/medicines/what-we-publish-medicines-when.
4. Goldstein DA, Chen Q, Ayer T, Howard DH, Lipscomb J, Harvey RD, et al. Cost Effectiveness Analysis of Pharmacokinetically-Guided 5-Fluorouracil in FOLFOX Chemotherapy for Metastatic Colorectal Cancer. Clin Colorectal Cancer. 2014; 13:219–25. http://doi.org/10.1016/j.clcc.2014.09.007. [PubMed]
5. Kawakami H, Zaanan A, Sinicrope FA. Implications of mismatch repair-deficient status on management of early stage colorectal cancer. J Gastrointest Oncol. 2015; 6(2):676–84. http://doi.org/10.3978/j.issn.2078-6891.2015.065. [PubMed]
6. von Borstel R, O'Neil JD, Saydoff JA, Bamat MK. Uridine triacetate for lethal 5-FU toxicity due to dihydropyrimidine dehydrogenase (DPD) deficiency. J Clin Oncol. 2010; 28:e13505–e13505. https://doi.org/10.1200/jco.2010.28.15_suppl.e13505.
7. Yajima S, Shimizu H, Sakamaki H, Ikeda S, Ikegami N, Murayama J. Real-world cost analysis of chemotherapy for colorectal cancer in Japan: detailed costs of various regimens during the entire course of chemotherapy. BMC Health Serv Res. 2016; 16:1–10. http://doi.org/10.1186/s12913-015-1253-x. [PubMed]
8. Palomaki GE, Bradley L a, Douglas MP, Kolor K, Dotson WD. Can UGT1A1 genotyping reduce morbidity and mortality in patients with metastatic colorectal cancer treated with irinotecan? An evidence-based review. Genet Med. 2009; 11:21–34. https://doi.org/10.1097/gim.0b013e31818efd77. [PubMed]
9. Srivastava, G, Renfro, LA, Behrens, RJ, Lopatin, M, Chao, C, Soori, GS, Dakhil, SR, Mowat, RB, Kuebler, JP, Kim, G, Mazurczak M. Prospective Multicenter Study of the Impact of Oncotype DX Colon Cancer Assay Results on Treatment Recommendations in Stage II Colon Cancer Patients. Oncologist. 2014; 19:492–7. https://doi.org/10.1634/theoncologist.2013-0401. [PubMed]
10. Lin J, Tan EC, Yang M. Comparing the effectiveness of capecitabine versus 5-fluorouracil / leucovorin therapy for elderly Taiwanese stage III colorectal cancer patients based on quality-of-life measures (QLQ-C30 and QLQ-CR38) and a new cost assessment tool. Health Qual Life Outcomes 2015; 13:61–70. https://doi.org/10.1186/s12955-015-0261-1. [PubMed]
11. National Institute for Health and Clinical Excellence - NICE. Cetuximab, bevacizumab and panitumumab for the treatment of metastatic colorectal cancer after first-line chemotherapy: Cetuximab (monotherapy or combination chemotherapy), bevacizumab (in combination with non-oxaliplatin chemotherapy) and panitumumab (monotherapy). Available 2019 Mar 11, from https://www.nice.org.uk/guidance/TA242/.
12. Lambrechts D, Lenz HJ, De Haas S, Carmeliet P, Scherer SJ. Markers of response for the antiangiogenic agent bevacizumab. J Clin Oncol. 2013; 3:1219–30. https://doi.org/10.1200/jco.2012.46.2762. [PubMed]
13. Therkildsen C, Bergmann TK, Henrichsen-Schnack T, Ladelund S, Nilbert M. The predictive value of KRAS, NRAS, BRAF, PIK3CA and PTEN for anti-EGFR treatment in metastatic colorectal cancer: A systematic review and meta-analysis. Acta Oncol. 2014; 53:852–64. https://doi.org/10.3109/0284186x.2014.895036. [PubMed]
14. National Institute for Health and Clinical Excellence - NICE. Nivolumab in combination with ipilimumab for treating advanced melanoma. Available 2019 Mar 13, from https://www.nice.org.uk/guidance/ta400/chapter/2-The-technology.
15. Toh JWT, de Souza P, Lim SH, Singh P, Chua W, Ng W, et al. The Potential Value of Immunotherapy in Colorectal Cancers: Review of the Evidence for Programmed Death-1 Inhibitor Therapy. Clin Colorectal Cancer. 2016; 15:285–91. http://doi.org/10.1016/j.clcc.2016.07.007. [PubMed]
16. National Institute for Health and Clinical Excellence - NICE. Aflibercept in combination with irinotecan and fluorouracil-based therapy for treating metastatic colorectal cancer that has progressed following prior oxaliplatin-based chemotherapy. Available 2019 Mar 13, from https://www.nice.org.uk/guidance/ta307.
17. Lambrechts D, Thienpont B, Thuillier V, Sagaert X, Moisse M, Peuteman G, et al. Evaluation of efficacy and safety markers in a phase II study of metastatic colorectal cancer treated with aflibercept in the first-line setting. Br J Cancer. 2015; 113:1027. https://doi.org/10.1038/bjc.2015.329. [PubMed]
18. National Institute for Health and Clinical Excellence - NICE. Ramucirumab for treating advanced gastric cancer or gastro–oesophageal junction adenocarcinoma previously treated with chemotherapy. Available 2019 Mar 14, from https://www.nice.org.uk/guidance/ta378/chapter/2-The-technology.
19. Zhu AX, Finn RS, Mulcahy M, Gurtler J, Sun W, Schwartz JD, et al. A phase II and biomarker study of ramucirumab, a human monoclonal antibody targeting the VEGF receptor-2, as first-line monotherapy in patients with advanced hepatocellular Cancer. Clin Cancer Res. 2013; 19:6614–23. https://doi.org/10.1158/1078-0432.ccr-13-1442. [PubMed]
20. National Institute for Health and Clinical Excellence - NICE. Regorafenib for metastatic colorectal cancer after treatment for metastatic disease (terminated appraisal). Available 2019 Mar 14, from https://www.nice.org.uk/guidance/ta334.
21. Furth E, Morrissette J, Deik AF, Loaiza-Bonilla A, Bonilla-Reyes PA, Shroff S, et al. KDR Mutation as a Novel Predictive Biomarker of Exceptional Response to Regorafenib in Metastatic Colorectal Cancer. Cureus. 2016; 8. https://doi.org/10.7759/cureus.478. [PubMed]
22. National Institute for Health and Clinical Excellence - NICE. Pembrolizumab for treating relapsed or refractory

classical Hodgkin lymphoma. Available 2019 Mar 14, from https://www.nice.org.uk/guidance/ta540/chapter/2-Information-about-pembrolizumab.

23. National Institute for Health and Clinical Excellence - NICE. Trifluridine–tipiracil for previously treated metastatic colorectal cancer. Available 2019 Mar 14, from https://www.nice.org.uk/guidance/TA405/chapter/2-The-technology/.
24. Behl AS, Goddard KAB, Flottemesch TJ, Veenstra D, Meenan RT, Lin JS, et al. Cost-Effectiveness Analysis of Screening for KRAS and BRAF Mutations in Metastatic Colorectal Cancer. J Natl Cancer Inst; 2012; 104:1785–95. http://doi.org/10.1093/jnci/djs433. [PubMed]
25. Medical Advisory Secretariat. KRAS Testing for Anti-EGFR Therapy in Advanced Colorectal Cancer. Ont Health Technol Assess Ser. 2010; 10:1–49. https://www.ncbi.nlm.nih.gov/pmc/articles/PMC3377508/?report=reader.
26. Shiroiwa T, Motoo Y, Tsutani K. Cost-Effectiveness Analysis of KRAS Testing and Cetuximab as Last-Line Therapy for Colorectal Cancer. Mol Diagn Ther. 2010; 14:375–84. http://doi.org/10.1007/bf03256395. [PubMed]
27. Vijayaraghavan A, Efrusy MB, Göke B, Kirchner T, Santas CC, Goldberg RM. Cost-effectiveness of KRAS testing in metastatic colorectal cancer patients in the United States and Germany. Int J Cancer. 2012; 131:438–45. https://doi.org/10.1002/ijc.26400. [PubMed]
28. Ayvaci MUS, Shi J, Alagoz O, Lubner SJ. Cost-Effectiveness of Adjuvant FOLFOX and 5FU/LV Chemotherapy for Patients with Stage II Colon Cancer. Med Decis Mak. 2013; 33:521–32. http://doi.org/10.1177/0272989x12470755. [PubMed]
29. Traoré S, Boisdron-Celle M, Hunault G, Andre T, Morel A, Guerin-Meyer V, et al. DPD deficiency: Medicoeconomic evaluation of pretreatment screening of 5-FU toxicity. J Clin Oncol. 2012; 30:410. http://doi.org/10.1200/jco.2012.30.4_suppl.410.
30. Tumeh, JW, Shenoy, PJ, Moore, SG, Kauh, J, Flowers C. A Markov model assessing the effectiveness and cost-effectiveness of FOLFOX compared with FOLFIRI for the initial treatment of metastatic colorectal cancer. Am J Clin Oncol. 2009; 32:49–55. https://doi.org/10.1097/coc.0b013e31817c6a4d. [PubMed]
31. Alberts SR, Yu TM, Behrens RJ, Renfro LA, Srivastava G, Soori GS, et al. Comparative Economics of a 12-Gene Assay for Predicting Risk of Recurrence in Stage II Colon Cancer. Pharmacoeconomics. 2014; 32:1231–43. http://doi.org/10.1007/s40273-014-0207-1. [PubMed]
32. Blank PR, Moch H, Szucs TD, Schwenkglenks M. KRAS and BRAF Mutation Analysis in Metastatic Colorectal Cancer: A Cost-effectiveness Analysis from a Swiss Perspective. Clin Cancer Res. 2011; 17:6338–46. http://doi.org/10.1158/1078-0432.ccr-10-2267. [PubMed]
33. Harty G, Jarrett J, Jofre-Bonet M. Consequences Of Biomarker Analysis On The Cost-Effectiveness Of Cetuximab In Combination With Irinotecan Based Chemotherapy For First-Line Treatment Of Metastatic Colorectal Cancer. Stratified Medicine At Work? Value Health. 2015; A456. https://doi.org/10.1016/j.jval.2015.09.1165. [PubMed]
